# 310. Antibiotic Duration and Risk of Treatment Failure in Acute Postoperative Spinal Implant Infections: A Multicenter Retrospective Cohort Study

**DOI:** 10.1093/ofid/ofaf695.106

**Published:** 2026-01-11

**Authors:** Don Bambino Geno Tai, Emily Poehlein, Jessica C O’Neil, Sandra B Nelson, Daniela F de Lima Corvino, Alaina Ritter, Jenny R Aronson, Laura Certain, Alexander M Tatara, Aaron J Tande, Daisuke Furukawa, Tarek Abdalla, Molly E Fleece, Oren Gottfried, Melissa Erickson, Cindy Green, Daniel G Tobert, Jessica Seidelman

**Affiliations:** University of Minnesota, Minneapolis, MN; Duke University, Durham, North Carolina; University of Pennsylvania, Philadelphia, Pennsylvania; Massachusetts General Hospital, Boston, MA; University of Alabama at Birmingham, Vestavia, AL; University of Florida, Gainesville, Florida; Stanford University, Stanford, California; University of Utah, Salt Lake City, Utah; University of Texas Southwestern Medical Center, Dallas, Texas; Mayo Clinic, Rochester, Minnesota; Stanford University, Stanford, California; University of Alabama at Birmingham, Vestavia, AL; University of Alabama at Birmingham, Vestavia, AL; Duke University, Durham, North Carolina; Duke University, Durham, North Carolina; Duke University, Durham, North Carolina; MGH, Boston, Massachusetts; Duke University School of Medicine, Durham, NC

## Abstract

**Background:**

Spinal implant infections (SII) are serious complications following instrumented spinal fusion. Debridement with implant retention requires antibiotic therapy, yet the optimal duration remains unclear. We assessed whether longer antibiotic duration is associated with reduced risk of treatment failure.

**Figure:**
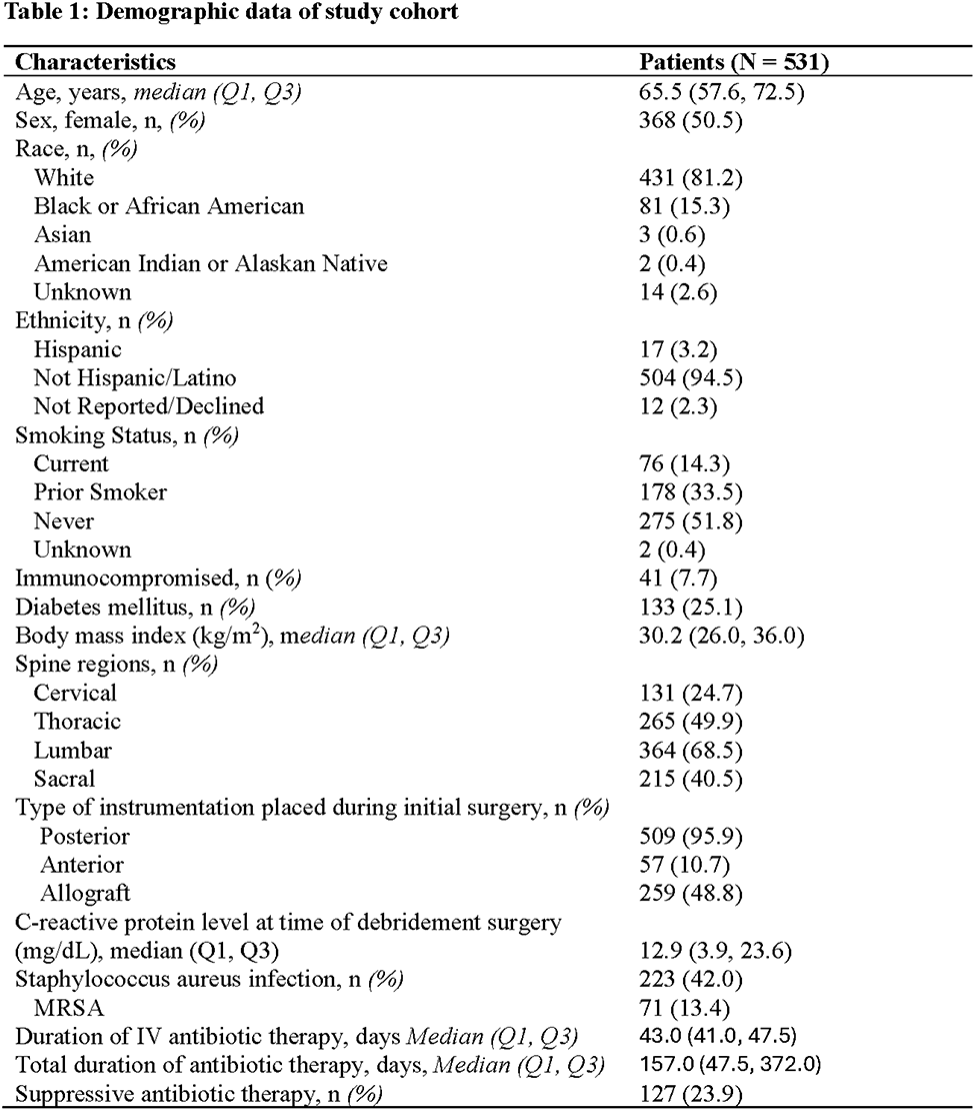

**Methods:**

We conducted a multicenter retrospective cohort study of adult patients diagnosed with acute postoperative SII (≤90 days from index surgery) between January 1, 2017, and December 31, 2022. All patients underwent debridement with implant retention. Treatment failure was defined as unplanned reoperation, clinical recurrence, or infection-related death. A Cox proportional hazards model assessed whether being on antibiotics was protective, treating antibiotic use as a time-dependent covariate, adjusting for relevant clinical variables, and including death not attributable to infection as a competing event. A second Cox model evaluated whether shorter antibiotic duration was associated with increased risk of failure with landmark analysis at six weeks.

**Results:**

There were 531 patients included in the study. Patient demographics are described in Table 1. During a median follow-up of 1,168 days (IQR 576-1,817), 151 patients experienced treatment failure. There was a non-significant trend toward decreased hazard of failure while on antibiotics (HR 0.70; 95% CI 0.46–1.05; p=0.087). Among 363 patients without early failure, 60 (16.5%) received ≤6 weeks of antibiotics and 303 (83.5%) received >6 weeks. Longer antibiotic duration was not associated with a differential risk of failure (HR 0.75, 95% CI 0.43–1.33; p=0.34).

**Conclusion:**

Continuing antibiotic therapy after debridement and implant retention may help reduce the risk of treatment failure in SII. However, it remains unclear whether extending antibiotic treatment beyond a certain duration continues to offer additional benefits. Prospective studies are needed to better define the optimal duration and approach to antibiotic therapy in these scenarios.

**Disclosures:**

Sandra B. Nelson, MD, UpToDate Wolters Kluwer Health: Author, Editor

